# Vitamin A fortification: key factors and considerations for effective implementation

**DOI:** 10.3389/fpubh.2025.1534375

**Published:** 2025-04-01

**Authors:** Joohun Han, Alvaro Durand-Morat, Khondoker Mottaleb

**Affiliations:** ^1^Department of Agricultural Economics and Agribusiness, University of Arkansas, Fayetteville, AR, United States; ^2^Department of Agricultural and Applied Economics, Texas Tech University, Lubbock, TX, United States

**Keywords:** food fortification, vitamin A, hidden hunger, food policy, policy design

## Abstract

Vitamin A fortification plays a crucial role in achieving long-term economic development in developing countries by supporting the growth and development of human capital. While fortification programs involve a range of nutritional, agricultural, economic, and political considerations, there is a lack of a comprehensive overview of the topic in the literature. Our review highlights the importance of holistic approach in designing fortification programs: the effective program should consider (a) the agronomic, economic, and administrative capability of the target regions in regard of fortification method and vehicle to maximize effectiveness; (b) strategies to ensure the producers' and consumers' adoption to enhance uptake rate; and (c) evaluate outcomes with respect to economic metrics rather than focusing solely on before-and-after comparison to avoid biased assessment.

## 1 Introduction

**Figure 1:**
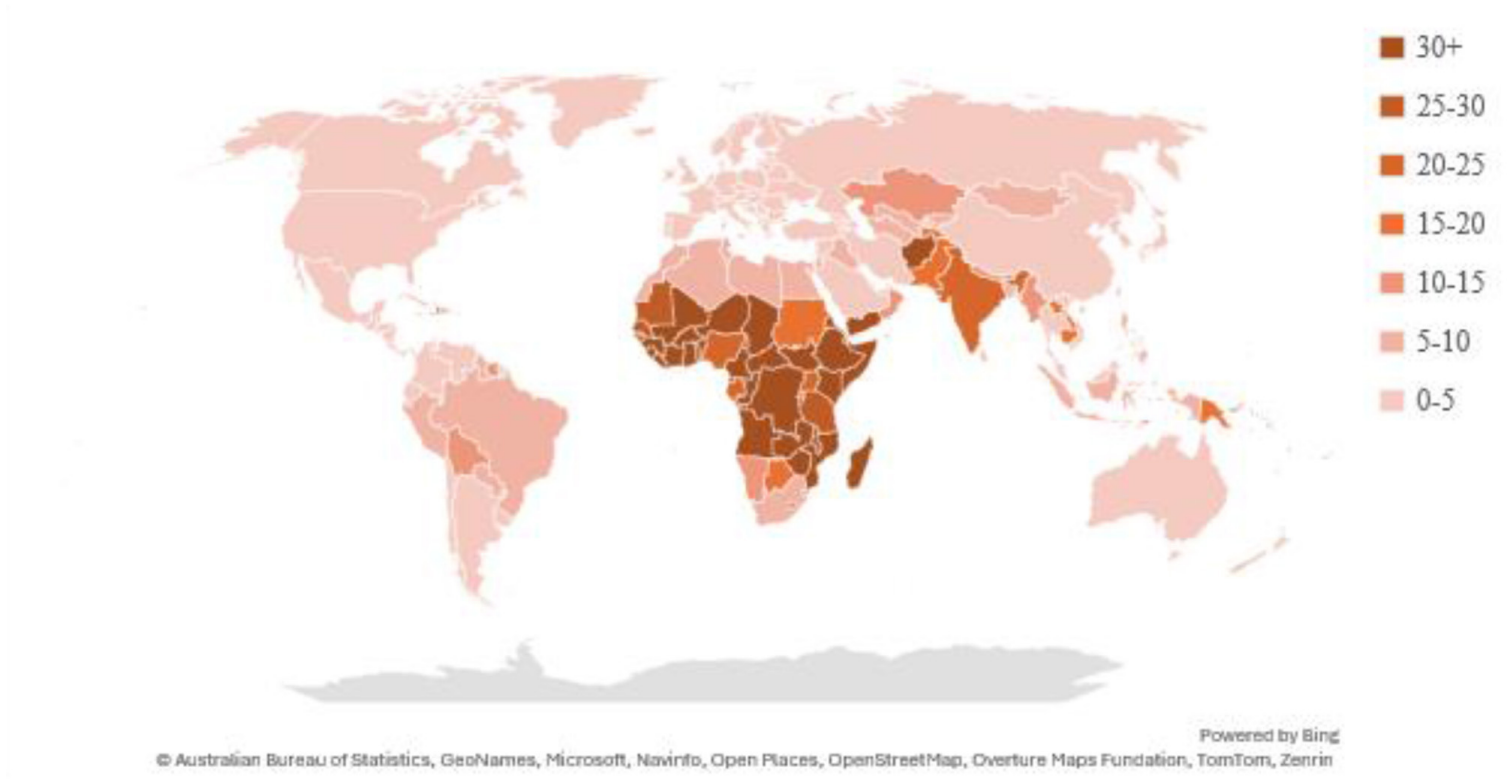
Age-standardized disability-adjusted life-year (DALY) rates per 100,000 by Vitamin A deficiency, 2021. Source: Institute For Health Metrics and Evaluation (4).

Hidden hunger (i.e., micronutrient deficiency) is a critical global problem affecting more than 2 billion people worldwide, impairing the cognitive and physical development of children and adolescents with long-term consequences on their livelihoods (1, 2). The most common micronutrient deficiencies globally are iodine, vitamin A, and iron (3). Vitamin A is a focal point in food fortification programs due to its significant public health implications. Vitamin A deficiency is a leading cause of vision impairment, childhood blindness, and maternal mortality, and has severe economic consequences by hampering labor productivity, raising healthcare expenses, and impeding workforce development, which worsens intergenerational poverty and economic inequality. Vitamin A deficiency is particularly prominent in developing countries in Africa and South Asia (Figure 1), where dietary options are often limited to starchy staples lacking vitamin A (5–8).

Among a few options, food fortification is an important tool to fight vitamin A deficiency, particularly for low-income households with limited access to nutritious foods. Food fortification refers to the deliberate increase in the amount of vitamins and minerals in food to improve its nutritional quality (8). Food fortification is often favored for its cost-effectiveness compared to alternatives such as supplementation (9). In 1922, Switzerland initiated the first food fortification program, adding iodine to salt to combat iodine deficiency disorders. Since then, many countries introduced fortification programs designed to their needs, yielding significant improvements in public health (1, 10).

Several organizations have developed vitamin A fortification programs through various pathways. Some programs focus on fortifying staple crops, while others focus on food additives (e.g., edible oil) and processed foods to ensure the target population meets their daily vitamin A requirements (11). Aside from a choice of fortification vehicles, several approaches are also available for fortifying food, such as biofortification (e.g., genetically modifying food crops or selected through conventional breeding programs to contain higher Vitamin A) and direct fortificant addition (e.g., adding vitamin A powder in processed foods). Each approach has pros and cons in terms of economic feasibility, nutritional efficiency, and sustainability. Also, the success rate of each method varies across different regions based on production (e.g., agronomic conditions) and demand (e.g., consumer socioeconomic conditions and preferences) factors (12).

From a public policy standpoint, it is important to analyze the factors that affect the performance of the fortification programs to aim the development of effective and efficient strategies for vitamin A fortification to address deficiency issues, improve public health outcomes, reduce healthcare costs, and enhance socioeconomic development in developing countries. This article provides an overview of ongoing vitamin A fortification programs across countries with the goal of informing interested stakeholders, including policymakers, about the factors to consider (e.g., supply, demand, infrastructure, etc.) for the design and administration of Vitamin A fortification programs.

## 2 Overview of food fortification programs

Food fortification aims to meet the recommended nutrition intake (RNI) of vitamin A for target population. According to World Health Organization (WHO) guideline, the RNI for Vitamin A is 500 μg for 19–50 year female, 600 μg for 19–50 years male, 400 μg for 1–3 years children, and 800 μg for pregnant women (13, 14). Selecting an appropriate fortification method and vehicle is crucial to ensure the RNI of target population, as different groups may have distinct dietary sources and food systems.

### 2.1 Fortification methods

The fortification methods can be broadly categorized as biofortification and conventional (i.e., direct or synthetic) fortification.

Biofortification is the process of enhancing the nutritional content of food crops and is considered an efficient approach to address undernourishment in rural areas in developing countries, where residents are predominantly self-sufficient farmers dependent on starchy staple crops and where inadequate market infrastructure and socioeconomic condition of the households hinders access to vitamin A-rich foods (9). Biofortification can be achieved through agronomic practices such as breeding. As of 2022, vitamin A-enriched banana/plantain, cassava, sweet potato, and maize have been developed and released in various countries (15). Conventional breeding is more accepted by consumers but limited fortification scope (restricted to fortification levels observed within the same specie), while genetic engineering offers a broader fortification scope (by allowing cross breeding with other species that show higher fortificant levels) but faces acceptance challenge (both from consumers and policymakers). For example, in 2004, the International Maize and Wheat Improvement Center (CIMMYT) initiated the vitamin A biofortified orange maize project through conventional breeding. As of 2022, orange maize has been released in 11 countries and is under field trial in 23 countries, effectively boosting the vitamin A intake of residents to meet their daily requirements (16). On the other hand, golden rice—a genetically modified rice enriched with beta carotene—has only been authorized for consumption in the Philippines more than two decades after its development (5). Thus, although biofortification is popular and proven effective for rural-dominant regions, gaining acceptance from farmers and consumers could be challenging, especially when produced using genetically engineered approaches.

Conventional fortification methods typically involve direct measures, such as dusting, diluting, or emulsifying vitamin A into food products. Conventional fortification may have cost and logistical advantages compared to biofortification in the short run because it does not require long-term research and development, and it is easier to control per unit dosage through mechanical approaches. Another advantage of conventional fortification is its flexibility; conventional fortification can be applied to a wide variety of food products and thus could be used on popular food products among the target population. For example, Thailand introduced vitamin A-fortified instant noodles in 1994. This initiative was significant given the widespread popularity of instant noodles among individuals, especially among low-income people (17). One of the limitations of this approach is the potential for over or underdose due to dietary change since conventional fortification typically achieves higher nutrient concentrations than biofortified sources (10, 18).

Each fortification approach has pros and cons (Table 1), and policymakers should consider these factors when designing vitamin A fortification programs.

**Table 1:** Advantages and drawbacks of fortification methods.

	**Pros**	**Cons**
Conventional fortification	Wide acceptance	Risk of over-fortification
	Controlled per unit dosage	Industrial constraints
	Cost-effectiveness in short-term	Sustainability concerns
Biofortification	Natural and sustainable	Longer development time
	Accessibility to rural areas	Geographic constraints
	Reduced dependence on processing	Uncertain acceptance and adoption

### 2.2 Choice of food vehicle

Selecting food items for fortification is another crucial aspect of a fortification program. A good vehicle for food fortification should be (a) affordable, (b) accessible, (c) acceptable, and (d) durable. The selected fortification vehicles need to be affordable and available for the target population, common or essential in the regional diet (a staple), and have a long shelf life without a refrigeration system.

**Figure 2:**
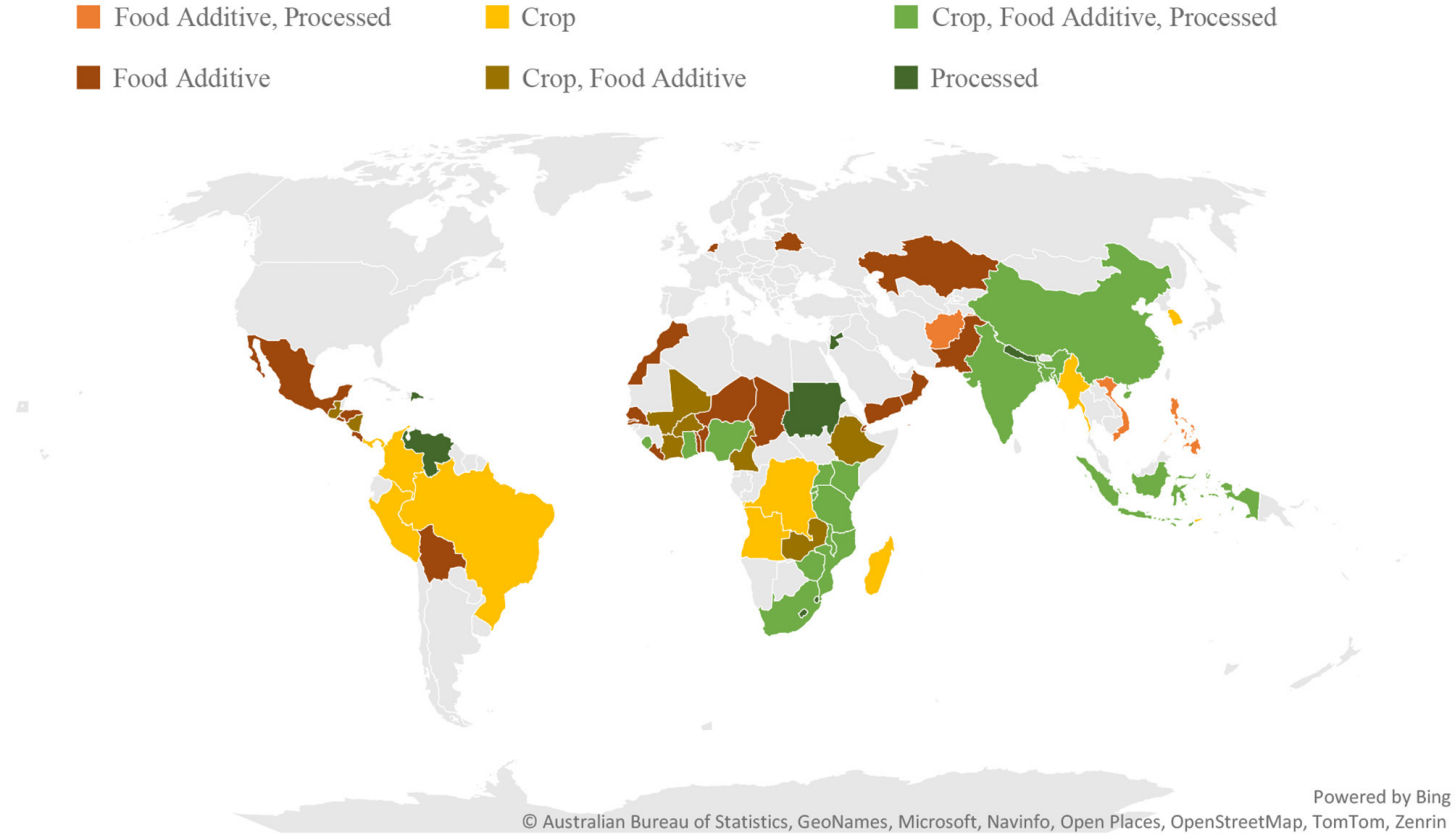
Vehicles for vitamin A fortification program by country: as of 2022. Sources: Arroyave and Mejia (21); Global Fortification (22); Guarnieri and Baral (15); Wirth et al. (11). The gray area indicates no data. This map does not account for supplementation (e.g., distributing vitamin pills) and pilot programs.

Considering these aspects, the commonly used food items for vitamin A fortification include grains, food additives, and processed foods that meet all four criteria. For instance, many countries in Central America, such as Guatemala, chose sugar as a conventional fortification vehicle because it is a commodity with an affordable price and high accessibility as the government promotes the sugar cane industry to generate employment (19). In Sub-Saharan Africa, such as Democratic Republic of the Congo, cassava and sweet potato are commonly selected as major vehicles for biofortification due to their long shelf-life and resilience to harsh agronomic conditions (15). In South Asia, such as Bangladesh and India, rice, wheat flour, and edible oil are utilized for conventional fortification vehicles due to their widespread production and consumption (20). As shown in Figure 2, most fortification programs are implemented in middle- and low-income countries where deficiencies are most prominent, and the selected food items vary by region.

Just like the fortification method, each food fortification vehicle has pros and cons. Grain crops are preferred in countries with high rural poverty as they are easily adjustable for local diets.

However, agronomic conditions could limit the application (e.g., maize may not be suitable for highland regions). Moreover, market conditions can also impact the choice of a vehicle. For example, despite rice being a primary staple in 49 developing countries with moderate to severe vitamin A deficiency issues, only six of these countries mandate rice fortification (2, 23). The market distortions created by domestic support and trade policies are cited as limitations to using rice as a vehicle (23). Moreover, conventional fortification methods require some industrialization throughout the supply chain (e.g., commercial mills), which does not exist in many settings where food processing is largely done on farms (e.g., rice parboiling in Burkina Faso).

Food additives such as edible oil and sugar are household staples, making them reliable for fortification programs. However, relying on food additives as a vehicle could lead to unhealthy dietary habits among consumers by promoting sugar and oil consumption. For example, the vitamin A-fortified sugar policy has demonstrated significant success in several countries (21, 24). However, reliance on a sugar-centric diet may induce a heightened risk of chronic diseases like diabetes, exacerbating health concerns for low-income households (18). Moreover, to enhance the effectiveness of programs, governments mandate the fortification of all sugar for local consumption. This may burden the sugar industry, requiring them to establish and oversee a fortification system differentiating non-fortified sugar for export (25, 26).

Processed food is a convenient and easily accessible vehicle for vitamin A fortification, appealing to a broad spectrum of consumers and offering a quick and effortless nutrient intake. For example, Pandesal, a type of bread made from vitamin A-fortified wheat flour, is a popular staple in the Philippines and has significantly increased the daily intake of vitamin A among children (27). However, despite its advantages, there are notable drawbacks to consider. Processed foods often contain high levels of sugar or salt, potentially contributing to unhealthy dietary habits (28). Furthermore, processing may reduce other nutritional aspects, such as processed wheat flour losing its fiber content. Fortification of processed foods assumes the target population can access markets to acquire these products. Therefore, its effectiveness is limited when the target population is isolated, which is the case for many rural households in developing countries.

Table 2 summarizes the discussions mentioned above. Thus, along with the fortification method, the food item should be carefully chosen based on agronomic conditions and the perception of the target population's fortification method.

**Table 2:** Advantages and drawbacks of each food item.

	**Pros**	**Cons**
Grain crop	Widely consumed staple food	Limited availability in certain regions
	Easily incorporated into existing diets	Limited by agricultural policy
	Sustainable solution	May alter taste, texture, and color
Food additive	Easy to implement and control dosage	May result in a regulatory burden
	Can be added to various food products	May raise concerns about health
	Provides flexibility in fortification	Potential for overconsumption
Processed food	Convenient and readily available	May contribute to unhealthy eating habits
	Appeals to a wide range of consumers	Processing may reduce nutrient content
	Provides quick and easy nutrient intake	Less effective in rural-prevalent regions

### 2.3 How can fortification programs benefit producers?

From the producers' perspective, participating in fortification programs involves risk with uncertain returns, as fortification can lead to physical or chemical changes (e.g., appearance, taste, aroma) that affect consumer acceptance (29, 30). To mitigate this issue, governments can apply follow-up policies that ensure producers' profit and encourage producers and consumers to adopt fortification programs. The common practice is follow-up training—such as instructions on cooking biofortified crops and education on their health benefits— for demand creation and encourage farmers to adopt biofortified crops for both self-consumption and sale, creating a stable market condition for biofortified crops (31). Other practices include mandating the use of conventional/bio-fortified foods in formal channels, like school meals, to promote their production and consumption (10, 31). Thus, such policies could enable the fortification program to address Vitamin A deficiency while also creating new market opportunities for producers.

### 2.4 Other factors to consider

In most cases, the reality on the ground is that vitamin A deficiency affects urban and rural households with varying degrees of income and access to markets, suggesting a mixed-method approach is needed to address the problem (9, 32). Most countries administer multiple fortification programs managed by entities ranging from international organizations such as UNICEF and CIMMYT to local institutes such as National Institute of Nutrition (NIN) in India and West African Health Organization (WAHO) in West Africa, ensuring cost efficiency and adequate vitamin A intake (33, 34). However, involving too many stakeholders may cause administrative and political hurdles that undermine the effectiveness of the interventions. Besides, applying for several fortification programs in the same region is prone to overlapping issues, potentially resulting in consumers overdosing on vitamin A (35).

For instance, Nigeria mandated the fortification of oil, sugar, and wheat and maize flours in 2000, 2002, and 2010, respectively, with vitamin A levels based on WHO recommendations. However, due to a lack of monitoring and evaluation efforts, the industry does not comply with the suggested level of fortification for those products (36). This poses a significant dilemma: while strict enforcement of fortification standards risks potential vitamin A overdosing, weak enforcement may continue the prevalence of vitamin A deficiency within the population. To overcome this issue, Friesen et al. (37) propose involving additional stakeholders to reform and oversee fortification programs, but this may require significant time and financial investment to be effective.

### 2.5 Program evaluation

Proper evaluation of fortification programs is essential for program design. In public policy, “effectiveness” refers a program's overall benefits, while “efficiency” assesses its cost relative to its impact (38).

Some common effectiveness measures include the Prevalence Ratio and total Disability-Adjusted Life-Year (DALY) averted. The Prevalence Ratio compares the rate of vitamin A deficiency in a specific area before and after the program (39), and provides a clear picture of the effectiveness of the program but ignores the effect of the intervention in other areas associated with vitamin A deficiency, such as healthcare cost savings or labor productivity gains. The DALY provides a broader assessment of the impact of a program by considering all possible health and productivity outcomes related to vitamin A deficiency. On the downside, estimating the DALYs is data intensive.

Efficiency is often assessed through the Cost-Benefit Ratio and cost per DALY averted. The Cost-Benefit Ratio compares costs and benefits in monetary terms but involves subjective valuation of outcomes (e.g., how to translate the saved lives into a monetary value). The cost per DALY averted offers a direct measure of cost-effectiveness by quantifying the cost per DALY averted.

The success of a fortification program depends on program specific factors as well as more general context conditions (e.g., human capital, infrastructure), and therefore cross-country comparisons must be carefully done. In general, developing countries may show lower effectiveness and efficiency than developed countries in absolute terms due to higher costs from limited infrastructure (40), yet vitamin A deficiency is more prevalent there. For example, Table 3 shows the total averted DALYs per year and the cost per DALY averted through folic acid fortification for neural tube defects (NTD) in the U.S., Chile, and Zambia. By comparing those three countries only with raw numbers, the U.S. appears as the most effective case (i.e., the most DALYs Averted) and Zambia as the most efficient case (i.e., the lowest Cost per DALY averted). However, when considering each country's population size and GDP, the narrative changes: Zambia shows the highest effectiveness (third column) and the lowest efficiency (fifth column) among those three countries. This highlights the need to consider factors such as population size and GDP when designing and evaluating a fortification program, especially in developing countries where effectiveness is crucial.

**Table 3:** Measuring Effectiveness and efficiency: the case of folic acid fortification in the U.S., Chile, and Zambia.

	**Effectiveness (higher is better)**		**Efficiency (lower is better)**	
	**Averted DALYs per year**	**Averted DALYs per year/total population (%)**	**Cost per DALY averted**	**Cost per DALY averted/GDP per capita (%)**
U.S.^a^	26,899	0.009	US$32.5	0.088
Chile^b^	2,500	0.016	US$89.0	0.790
Zambia^c^	17,286	0.174	US$14.9	4.093

^a^As of 2000, based on Bentley et al. (41)'s estimation with 700 μg/100 g case.

^b^As of 2001, based on Llanos et al. (42)'s estimation (no dosage information is disclosed).

^c^As of 2000, based on Hoddinott (43)'s estimation (no dosage information is disclosed).

The political and administrative feasibilities of the target region also need to be considered when evaluating the program. For example, the major vehicle for vitamin A fortification in Uganda is vegetable oil, which is mainly imported, despite the evidence that sugar, a major crop in Uganda, could be a suitable vehicle. Uganda's sugar sector has not embraced fortification as they perceive it as a risk (e.g., changes in quality could affect consumption) (13, 30). As a result, the estimated cost per DALY averted by sugar is 5-fold that of vegetable oil in Uganda, even though vegetable oil is import-based and sugar is locally produced.

## 3 Policy recommendation and conclusions

Effective vitamin A fortification programs can play a crucial role in improving public health outcomes, reducing malnutrition-related issues, and preventing human capital loss. To effectively address vitamin A deficiency through fortification programs, policymakers should adopt a multifaceted approach that considers the unique agronomic, economic, and social contexts of the target regions.

### 3.1 Fortification method and its vehicle

A mixed-methods approach that combines both biofortification and conventional fortification methods is recommended, especially in areas with diverse agricultural and socioeconomic statuses. Biofortification should be prioritized in rural areas where the population relies on staple crops, and market access is limited, while conventional fortification methods, such as fortifying widely consumed food products like edible oil or sugar, can be implemented in urban settings.

Additionally, the choice of fortification vehicle is critical; selecting foods that are affordable, accessible, and culturally accepted by the target population will enhance program uptake. For example, countries such as Somalia and Niger, where vitamin A deficiency is prevalent, can benefit from targeted biofortification efforts using appropriate vehicles like sorghum, which is a locally produced and consumed staple capable of thriving in challenging agronomic conditions (22, 44, 45).

### 3.2 Fortification program design

To ensure the success of fortification programs, governments must encourage local producers to participate by providing adequate support, such as subsidies, training, and a clear regulatory framework. This support should also address potential market distortions and ensure that producers can participate sustainably. Strong monitoring and evaluation systems are crucial to preventing issues like over-fortification or under-fortification, ensuring that the levels of vitamin A added to food products meet the nutritional needs of the population.

Engaging local stakeholders—including agricultural producers, food processors, and consumers—is essential for the acceptance and sustainability of fortification programs. Involvement of local communities helps tailor interventions to cultural preferences and practices, ensuring broader program adoption. Additionally, thorough evaluation of existing fortification programs is necessary to assess their effectiveness in addressing vitamin A deficiency and optimize them to meet the unique challenges faced by different regions.

Finally, the long-term success of vitamin A fortification programs depends on their continuous adaptation to the specific needs and resources of each region. Regular assessments of program effectiveness, alongside ongoing stakeholder engagement, will ensure that these programs deliver tangible health benefits, contributing to the reduction of vitamin A deficiency and the improvement of public health outcomes across diverse populations.
